# The Origins of Individual Differences in How Learning Is Expressed in Rats: A General-Process Perspective

**DOI:** 10.1037/xan0000116

**Published:** 2016-10

**Authors:** E. Patitucci, A. J. D. Nelson, Dominic M. Dwyer, R. C. Honey

**Affiliations:** 1 School of Psychology and Education Sciences, University of Bologna; 2 School of Psychology, Cardiff University

**Keywords:** sign-tracking, goal-tracking, reinforcer value, palatability, devaluation

## Abstract

Laboratory rats can exhibit marked, qualitative individual differences in the form of acquired behaviors. For example, when exposed to a signal-reinforcer relationship some rats show marked and consistent changes in sign-tracking (interacting with the signal; e.g., a lever) and others show marked and consistent changes in goal-tracking (interacting with the location of the predicted reinforcer; e.g., the food well). Here, stable individual differences in rats’ sign-tracking and goal-tracking emerged over the course of training, but these differences did not generalize across different signal-reinforcer relationships (Experiment 1). This selectivity suggests that individual differences in sign- and goal-tracking reflect differences in the value placed on individual reinforcers. Two findings provide direct support for this interpretation: the palatability of a reinforcer (as measured by an analysis of lick-cluster size) was positively correlated with goal-tracking (and negatively correlated with sign-tracking); and sating rats with a reinforcer affected goal-tracking but not sign-tracking (Experiment 2). These results indicate that the observed individual differences in sign- and goal-tracking behavior arise from the interaction between the palatability or value of the reinforcer and processes of association as opposed to dispositional differences (e.g., in sensory processes, “temperament,” or response repertoire).

Individual differences in human behavior reflect the interplay between genetic and environmental factors (e.g., Plomin, 1994). The restricted genetic background and shared environments of many laboratory rats might lead one to predict that individual differences in their adaptive behavior will be limited; and dominant models of animal learning eschew consideration of such differences (e.g., Rescorla & Wagner, 1972). However, laboratory rats that have received simple Pavlovian conditioning, where the temporary presentation of a lever signals a food reinforcer, express the fact that they have detected this relationship in very distinct ways: some by interacting with the signal (called sign-tracking) and others by approaching the site of food delivery (called goal-tracking; e.g., Fitzpatrick et al., 2013). The potential similarities between Pavlovian sign-tracking behaviors and those elicited by drugs of abuse have long been noted (for a review, see Tomie, Grimes, & Pohorecky, 2008), which has led some to argue that sign-tracking represents a behavioral marker for components of substance abuse (e.g., Flagel, Akil, & Robinson, 2009). This argument is consistent with the neurobiological analysis suggesting that sign-tracking (but not goal-tracking) is linked to mesolimbic dopamine activity (Flagel et al., 2011; see also Robinson & Berridge, 1993). The attraction of rats to the signs that a reinforcer is imminent has been aligned to processes that might support both the development and maintenance of substance abuse. However, the tendency for rats to approach the site in which the reinforcer will be delivered during the lever is also relevant to the development of substance abuse: visiting locations where reinforcers are available is also a component of drug seeking. While the differences in the behavioral expression of learning have translational relevance, they also have broad theoretical significance. For example, they have significance in the context of attempts to build general-process (associative) models of learning.

Pavlov (1935) saw behavioral conditioning as the missing link between conceptual and neurobiological analyses of learning. However, the qualitative individual differences in how learning is expressed that were described above (e.g., Fitzpatrick et al., 2013) are left unexplained by associative models of Pavlovian learning (e.g., Mackintosh, 1975; Pearce & Hall, 1980; Rescorla & Wagner, 1972). According to such models, the predictive relationship between the lever and the delivery of food should result in the formation of a link or association between the representations of the two allowing future presentations of the lever to activate a memory of food. This analysis allows that the exact form of conditioned response might not mimic the specific nature of the response to food itself (Wagner & Brandon, 1989), but they offer no coherent account for individual differences in the form of conditioned behavior. This omission is perhaps unsurprising given the fact that these general-process accounts were not intended to provide a characterization of individual differences. Moreover, it remains possible for them to appeal to variation in a set of very general processes in explaining differences in the form of the conditioned response across individuals (cf. Matzel et al., 2003). For example, variations in sensory processes, general states (arousal, anxiety, or general motivation) or behavioral repertoire per se might each provide a basis for whether rats will become sign-trackers or goal-trackers: a rat might be more inclined to sign track to the extent that the signal is processed effectively, provokes arousal, or the rat has a propensity to explore. However, if it proved that the individual differences under consideration were a function of how the reinforcer is processed, something that is integral to the process of associative learning (e.g., Rescorla & Wagner, 1972), then these associative models would need to provide an internally consistent analysis for them.

While there has been increasing interest in examining the neurobiological correlates of sign- and goal-tracking, behavioral analysis remains more limited. The two experiments described here examined the origins of sign- and goal-tracking through such a behavioral analysis. In Experiment 1, rats received separate trials on which the presentation of one lever (e.g., the left lever) signaled the delivery of food pellets and another lever (e.g., the right lever) signaled the delivery of sucrose. As we have already noted, previous research indicates that temporally stable individual differences in sign- and goal-tracking should emerge in the presence of the levers (e.g., Fitzpatrick et al., 2013). The issue of primary interest was whether these differences would be consistent across different signal-reinforcer relationships, or more selective and potentially based on individual differences in the value placed on specific reinforcers: Would individual differences in sign- or goal-tracking on the left lever (signaling one reinforcer) be related to how rats responded to the right lever (signaling another reinforcer); or would individual differences on one lever be unrelated to behavior on the other lever? This issue has not been explored in previous studies, which have examined sign-tracking to multiple signals paired with different reinforcers, but under conditions in which sign-tracking was the sole measure of performance, because the reinforcers were intraorally infused (Robinson & Berridge, 2013). Experiment 2 assessed whether or not individual differences in sign- and goal-tracking were related to the palatability of (or liking for) a reinforcer. To do so, two strategies were adopted. First, sessions in which we assessed the palatability of sucrose through a microstructural analysis of licking (e.g., Davis & Smith, 1992; Dwyer, 2012) were interleaved with sessions where the presentation of a lever signaled the delivery of sucrose (and another lever did not). In this case, the relationship between palatability and behavioral expression of learning (sign- or goal-tracking) was assessed. Second, after the completion of training, the influence of changes in reinforcer value on sign- and goal-tracking was assessed by sating rats with sucrose before a test in which the levers were presented, but no sucrose was delivered. Previous studies that have examined the effects of this manipulation have done so in a ways that yield results that are difficult to interpret: Because all rats were sated with a reinforcer before the test, changes in sign- and goal-tracking during the test (relative to baseline) could have a number of nonspecific origins; especially given the fact that these behaviors were not under discriminative control (Cleland & Davey, 1982; see also, Davey & Cleland, 1984). Moreover, there is some evidence that goal-tracking rats become less likely to goal track, and more likely to sign track, after the reinforcer (in this case sucrose) had been devalued by pairing it with lithium chloride (Morrison, Bamkole, & Nicola, 2015).

## Experiment 1

The experimental design used in Experiment 1 was simple. Food-restricted rats were placed in an operant chamber where the insertion of a retractable lever to the left of a food well signaled the delivery of one reinforcer (e.g., food pellets) whereas the insertion of a second lever to the right of the well signaled the delivery of a second reinforcer (e.g., sucrose) to the same well. The rats’ interactions with the levers (sign-tracking) and their entries into the well (goal-tracking) during the presentation of the levers were automatically recorded. The issue of principal interest was whether individual differences in sign-tracking or goal-tracking would be general (evident during presentations of both levers) or specific (evident for one lever, but not the other).

To assess individual differences, we trained the group of rats until they had reached a stable level of performance (in terms of the number of lever presses and food-well entries) on both levers. We then separated the rats on the basis of their tendency to engage in sign- or goal-tracking behaviors at the end of training. This was achieved by calculating a bias score: (number goal-tracking responses—number of sign-tracking responses)/(number goal-tracking responses + number of sign-tracking responses), and then taking a median split. Using this score a sign-tracking bias is indicated by a negative score and a goal-tracking bias by a positive score. Critically, we performed this split twice: once based on responses during the left lever and once based on responses during the right lever. If the tendency to sign- or goal-track on a given lever is reliable then these classifications should remain stable across blocks of trials. More important, if the tendency to sign- or goal-track entirely reflects differences in sensory processes, general affective states, behavioral repertoire per se, or to variations in a neurotransmitter system, then the classifications should also be stable across the two levers. Thus, we first examined the consistency of the bias in these subgroups across trial blocks for a given lever; and then examined the consistency of the bias within a trial block, but critically across the two levers. Using the subgroups created at the end of training, we also examined the development of both sign- and goal-tracking across all blocks of training separately for both the left and right levers.

The form of analysis described above classifies rats into groups based on similar sign- or goal-tracking biases. It is convenient and enables the development of sign- and goal-tracking behaviors to be tracked and readily visualized across the sessions up to and including the terminal sessions that formed the basis of the classification. However, this analysis has several well-rehearsed limitations: it necessarily treats individual differences as categorical rather than continuous, and in doing so loses potential power. Therefore, we also approached the data in a complementary fashion, treating individual differences as a continuous variable. Using this analysis, consistent individual differences in sign-tracking (or goal-tracking) on one lever would be evident as a correlation between the bias scores on that lever during block *n* and block *n* + 1. Of greater potential theoretical significance is whether or not the bias scores on the two levers correlated with one another within a given trial block.

### Method

#### Subjects

Thirty-two naïve male (outbred) Lister Hooded rats (supplied by Harlan Laboratories, United Kingdom) were housed in pairs in standard cages and maintained on 12-hr/12-hr light/dark cycle (lights on at 7 a.m.). Their mean ad libitum weight was 308 g (range: 285−327 g). Rats had free access to water and they were maintained between 85 and 90% of their ad lib weights by giving them restricted access to food at the end of each day.

#### Apparatus

The experimental room contained eight identical conditioning boxes measuring 30 × 24 × 21 cm (H × W × D; Med Associates, Georgia, VT) that were placed in sound-attenuating shells. The ventilation fan for each shell maintained the background noise at 68 dB. The boxes had aluminum front and back walls and clear acrylic sides and top. The floor was constructed from 19 steel rods: 4.8 mm diameter, 16 mm apart situated above a stainless tray. The food pellets (45 mg: supplied by MLab: Richmond, IN) and sucrose solution (8%/water; from a dipper) could be separately delivered to a recessed food well equipped with infrared detectors located in the center of the left wall. Two retractable levers were located 3 cm to the left and right of the food well (4.6 × 1.5 cm), which was centrally located in the same wall, but at floor level. MED-PC software was used to insert levers, deliver stimuli, and to record food well entries and lever presses. Food well entries were assessed using a system of photo beams across the entrance, and a lever press was recorded on each occasion that the lever was depressed by 4 mm from its usual resting position.

#### Procedure

##### Pretraining

Rats had two 24-min pretraining sessions. In the first session, half of the rats were trained to consume the sucrose from the food wells in the conditioning boxes and the remaining rats were trained to consume food pellets. In the second session, the rats given sucrose in the first session received food pellets and vice versa. During both sessions, the reinforcers were delivered on a variable-time (VT) 60-s schedule (range: 40–80 s).

##### Conditioning

Rats received 12 days of conditioning that occurred at the same time of day for a given rat. On each day, there was a single session that consisted of 20 trials where the left lever was inserted into the box for 10 s and was then retracted, and 20 trials where the right lever was inserted for 10 s and then retracted. For half of the rats, the retraction of the left lever was immediately followed by the dipper being immersed in the sucrose and then raised back into the food well, and the retraction of the right lever was immediately followed by the delivery of one food pellet. For the remaining rats this arrangement was reversed. The two trial types were presented randomly with the constraint that there were no more than three trials of the same type in succession. The trials were delivered according to a VT 60-s (range: 40–80 s) schedule.

### Data Analysis

In Experiment 1, the absence of a relationship (e.g., between the bias score on the two levers) and the presence of a relationship are both of potential theoretical significance. Standard null-hypothesis significance testing only assesses how unlikely the observed data is given the assumption of the null hypothesis. It does not directly assess whether the absence of a significant effect provides good evidence for there being no true relationship. In contrast, Bayesian tests are based on calculating the relative probabilities of the null and alternative hypotheses, and thus, afford an assessment of whether the evidence is in favor of either hypothesis. The Bayes factor relates to the ratio of probability for the observed data under a model based on the null hypothesis compared with a model based on some specified alternative model. Bayes factors can be denoted as B_01_ when the data support the null, or B_10_ when the data support the alternative. The resulting Bayes factors can then be interpreted according to the convention suggested by Jeffreys (1961) and recommended by Rouder, Speckman, Sun, Morey, and Iverson (2009) and by Robert, Chopin, and Rousseau (2009): a Bayes factor between 1 and 3 gives anecdotal support, a factor between 3 and 10 suggests some supporting evidence, while a factor beyond 10 indicates strong evidence. We have, therefore, supplemented the report of “standard” null hypothesis tests with the presentation of equivalent Bayes factors where appropriate; and in particular where null results are of theoretical significance. These were calculated using JASP 0.7.1.12 (Love et al., 2015).

### Results

The 12 training sessions were combined into six consecutive 2-day blocks. For each of the final two blocks, bias scores were calculated separately for the left and right levers: (number goal-tracking responses—number of sign-tracking responses)/(number goal-tracking responses + number of sign-tracking responses). As already noted, a sign-tracking bias is indicated by a negative score and a goal-tracking bias by a positive score. Critically, we performed this analysis separately for behavior on the left and right levers. These scores were then subject to a median split that created subgroups reflecting sign-tracking (low bias score) and goal-tracking (high bias scores).

Table 1 shows the number of rats classified as sign-tracking or goal-tracking. Inspection of Sections A and B shows that classifications were highly consistent between Blocks 5 and 6 for the left lever (χ^2^ (1) = 12.5, *p* < .001, B_10_ = 113^1^) and the right lever (χ^2^ (1) = 18.0, *p* < .001, B_10_ > 1,000). In contrast, inspection of Sections C and D shows that classification as sign- or goal-tracking on the left and right levers was independent for both Block 5 (χ^2^ (1) = 0.5, *p* = .480, B_01_ = 3.7) and Block 6 (χ^2^ (1) = 0, *p* > .999, B_01_ = 4.7). That is, the tendency of rats to be biased toward sign- or goal-tracking on one lever (paired with one reinforcer) was unrelated to their propensity to be biased toward sign- or goal-tracking on the other lever (paired with a second reinforcer).[Table-anchor tbl1]

**Figure 1 fig1:**
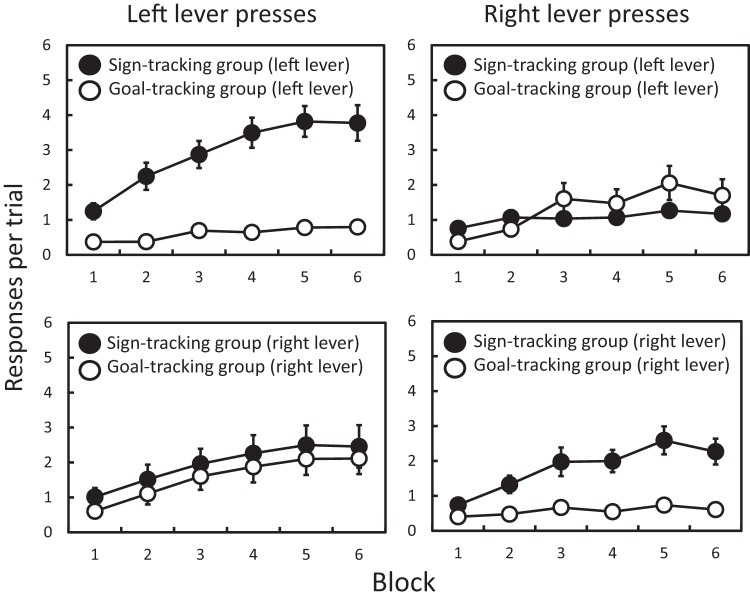
Experiment 1. Mean (±*SEM*) lever press responses per trial, across two-session blocks. The left-hand panels show left lever presses for groups that had been classified on the basis of their sign- versus goal-tracking bias on the left lever (upper panel) or right lever (lower panel). Similarly, the right-hand panels shows right lever presses for groups that had been classified on the basis of their sign- versus goal-tracking bias on the right lever (lower panel) or left lever (upper panel). The classifications were made independently on the biases to sign- or goal-track for the left lever (upper panels) and right lever (lower panels) that were paired separately with different reinforcers (food pellets and sucrose) in a counterbalanced manner.

**Figure 2 fig2:**
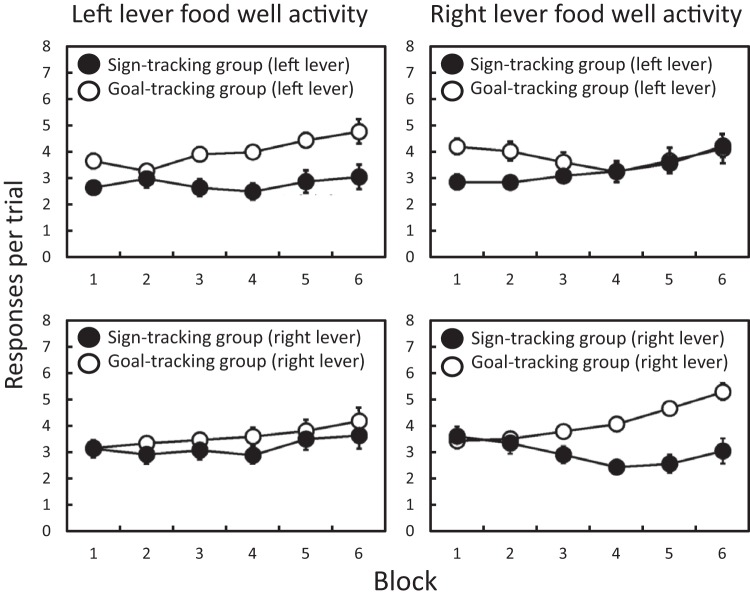
Experiment 1. Mean (±*SEM*) food well entries per trial in Experiment 1, across two-session blocks. The left-hand panels shows food well entries during the left lever for groups that had been classified on the basis of their sign- versus goal-tracking bias on the left lever (upper panel) or right lever (lower panel). Similarly, the right-hand panels shows food well entries during the right lever for groups that had been classified on the basis of their sign- versus goal-tracking bias on the right lever (lower panel) and left lever (upper panel). The classifications were made independently on the biases to sign- or goal-track for the left lever (upper panels) and right lever (lower panels) that were paired separately with different reinforcers (food pellets and sucrose) in a counterbalanced manner.

The development of independent sign- and goal-tracking behavior on the two levers is illustrated in Figure 1 (showing lever press responses) and Figure 2 (showing food well entries); with the upper two panels in each figure representing the data split by the sign- versus goal-tracking classification on the left lever (taken across Blocks 5 and 6); and the lower two panels in each figure representing the data split by the sign- versus goal-tracking classification on the right lever. Inspection of these figures suggests that: when the classification is done for the left lever, the sign- and goal-tracking behaviors emerge across training for the left lever, but not for the right lever; while when the classification is done for the right lever, the sign- and goal-tracking behaviors emerge across training for the right lever, but not for the left lever. These results were analyzed using mixed analysis of variances (ANOVAs) with repeated measures factors of training block and lever (left versus right), and a between-subjects factor of sign- versus goal-tracking classification. The analysis was performed separately for lever press responses and food well entries, and for classification on the left and right lever.[Fig-anchor fig1][Fig-anchor fig2]

For lever press responses, with groups classified on the basis of the left lever (upper panels of Figure 1), the ANOVA revealed significant main effects of block (*F*(5, 150) = 30.88, *p* < .001, η_p_^2^ = .507), lever (*F*(1, 30) = 5.62, *p* = .024, η_p_^2^ = .158), and sign- versus goal-tracking classification (*F*(1, 30) = 13.19, *p* = .001, η_p_^2^ = .305). There was no block-by-classification interaction (*F*(5, 150) = 1.942, *p* = .091, η_p_^2^ = .061) or lever-by-block interaction (*F*(5, 150) = 2.12, *p* = .056, η_p_^2^ = .069). Critically, for the idea that classification on the left lever selectively reflects behavior on that lever, there were significant interactions between sign- versus goal-tracking classification and lever (*F*(1, 30) = 29.80, *p* < .001, η_p_^2^ = .490), and a significant three-way interaction (*F*(5, 150) = 16.31, *p* < .001, η_p_^2^ = .352). The critical theoretical result relating to the independence of sign- and goal-tracking behavior between levers is whether assigning rats to sign- or goal-tracking groups on the basis of one lever, predicts their performance on the other lever: that is, the interaction between sign- versus goal-tracking classification and lever. Therefore, to assess the robustness of the standard ANOVA approach, Bayesian ANOVAs were also calculated and the model based on the interaction was compared to the best model without the interaction. This analysis gives a Bayes factor of more than 1,000 in favor of the model with the interaction.^2^

For lever press responses, with groups classified on the basis of the right lever (lower panels of Figure 1), the ANOVA revealed significant main effects of block (*F*(5, 150) = 31.34, *p* < .001, η_p_^2^ = .511) and sign- versus goal-tracking classification (*F*(1, 30) = 7.28, *p* = .011, η_p_^2^ = .195), but no effect of lever (*F*(1, 30) = 3.04, *p* = .092, η_p_^2^ = .092). There was a block-by-classification interaction (*F*(5, 150) = 2.42, *p* = .038, η_p_^2^ = .075), but no lever-by-block interaction (*F*(5, 150) = 1.54, *p* = .180, η_p_^2^ = .049). Although there was no interaction between sign- versus goal-tracking classification and lever (*F*(1, 30) = 1.75, *p* = .196, η_p_^2^ = .055), the three-way interaction did approach the conventional level of statistical significance (*F*(5, 150) = 2.59, *p* = .051, η_p_^2^ = .070). Moreover, a Bayesian ANOVA comparison of the model based on the interaction to the best model without the interaction gives a Bayes factor of 28.1 in favor of the model with the interaction.

For food well entries, with groups classified on the basis of the left lever (upper panels of Figure 2), the ANOVA revealed significant main effects of block (*F*(5, 150) = 5.09, *p* < .001, η_p_^2^ = .145) and sign- versus goal-tracking classification (*F*(1, 30) = 7.12, *p* = .012, η_p_^2^ = .192), but not of lever (*F*(1, 30) = 0.78, *p* = .383, η_p_^2^ = .025). There was no block-by-classification interaction (*F*(5, 150) = 0.36, *p* = .878, η_p_^2^ = .012) or block-by-lever interaction (*F*(5, 150) = 0.59, *p* = .707, η_p_^2^ = .019). While there was no significant interaction between sign- versus goal-tracking classification and lever (*F*(1, 30) = 3.79, *p* = .061, η_p_^2^ = .112), there was a significant three-way interaction (*F*(5, 150) = 6.12, *p* < .001, η_p_^2^ = .170). A Bayesian ANOVA comparison of the model based on the interaction to the best model without the interaction gives a Bayes factor of 3.3 in favor of the model with the interaction.

For food well entries, with groups classified on the basis of the right lever (lower panels of Figure 2), the ANOVA revealed significant main effects of block (*F*(5, 150) = 5.91, *p* < .001, η_p_^2^ = .144) and sign- versus goal-tracking classification (*F*(1, 30) = 5.41, *p* = .027, η_p_^2^ = .153), but not of lever (*F*(1, 30) = 0.79, *p* = .382, η_p_^2^ = .026). There was a block-by-classification interaction (*F*(5, 150) = 5.23, *p* < .001, η_p_^2^ = .149), but no block-by-lever interaction (*F*(5, 150) = 0.55, *p* = .737, η_p_^2^ = .018). Although there was no interaction between sign- versus goal-tracking classification and lever (*F*(1, 30) = 4.01, *p* = .053, η_p_^2^ = .119), there was a significant three-way interaction (*F*(5, 150) = 3.69, *p* = .004, η_p_^2^ = .110). A Bayesian ANOVA comparison of the model based on the interaction to the best model without the interaction gives a Bayes factor of 4.6 in favor of the model with the interaction.

**Figure 3 fig3:**
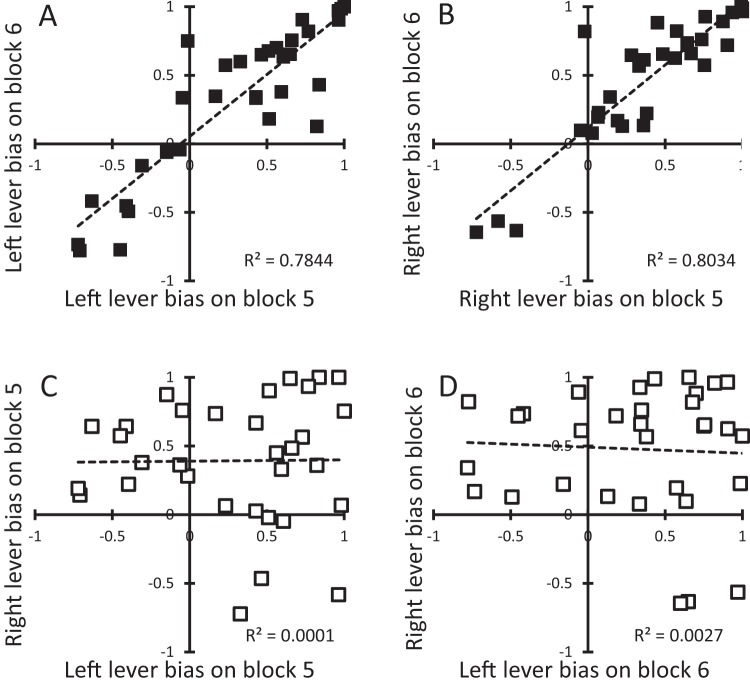
Experiment 1. Relationships between: A = sign- versus goal-tracking bias on Blocks 5 and 6 for the left lever; B = sign- versus goal-tracking bias on Blocks 5 and 6 for the right lever; C = sign- versus goal-tracking bias for the left versus right lever on Block 5; D = sign- versus goal-tracking bias for the left versus right lever on Block 6. A sign-tracking bias is evident as a negative value and a goal-training bias as a positive value; and the left and right levers were paired separately with food pellets or sucrose in a counterbalanced manner.

Turning to the group-level correlations, the upper two panels of Figure 3 show the relationship between bias scores on a single lever (with the left and right levers treated separately) for Blocks 5 and 6. There were clear positive correlations for both the left lever (*r* = .89, *p* < .001, B_10_ > 1,000) and right lever (*r* = .90, *p* < .001, B_10_ > 1,000), indicating that the tendency to show a bias toward sign- or goal-tracking behavior was consistent—within a single lever—across Blocks 5 and 6. The lower two panels of Figure 3 show the relationship between bias scores on the left and right levers within a block (with Blocks 5 and 6 treated separately). There was no clear relationship between bias scores on one lever and those on the other lever for either Block 5 (*r* = .01, *p* = .875, B_01_ = 4.5) or Block 6 (*r* = −.05, *p* = .777, B_01_ = 4.3).[Fig-anchor fig3]

### Discussion

The results of Experiment 1 confirm that our procedure produces marked individual differences in sign-tracking and goal-tracking. The unique feature of the results is that these differences were specific to a given signal-reinforcer relationship: sign-tracking on the left lever (paired with one reinforcer) was unrelated to sign-tracking on the right lever (paired with another reinforcer) and goal-tracking during the left lever was unrelated to goal-tracking on the right lever. This was true whether sign- versus goal-tracking was treated as a categorical difference (see the analysis of the data presented in Table 1, and in Figures 1 and 2) or as a continuous variable (see the analysis of the data presented in Figure 3). One interpretation of the specific nature of individual differences in sign- and goal-tracking is that they reflected individual differences in the value of the two reinforcers. For example, a rat that values sucrose might be more likely to show marked sign-tracking (or goal-tracking) on the lever that signals sucrose, but not the lever that signals food. Experiment 2 assessed this interpretation directly.

## Experiment 2

In Experiment 2, rats received cycles of two types of session. In the first type, they were placed in a chamber where their consumption of sucrose was measured, and the microstructure of their licking patterns was assessed. Lick-cluster size was used as a measure of palatability or liking, with higher lick cluster sizes indicating higher palatability or liking. The grounds for the use of this measure were as follows. Analysis of the microstructure of rats’ licking behavior reveals that they typically produce rhythmic sets of licks that group into clusters separated by pauses. The mean number of licks in a cluster (called cluster size) is directly related to the concentration of palatable and unpalatable solutions independently of the amount consumed, with palatable solutions resulting in larger lick cluster sizes than unpalatable ones (for a review see, Dwyer, 2012; see also Davis & Perez, 1993; Davis & Smith, 1992; Spector, Klumpp, & Kaplan, 1998). Moreover, there is a double-dissociation where some manipulations influence lick cluster size but not overall consumption (e.g., Dwyer, 2008) while others influence consumption but not lick cluster size (e.g., Dwyer, Boakes, & Hayward, 2008). These relationships imply that that lick cluster size provides an index of the hedonic value of the solution being consumed that is independent of total intake measures.

In the second type of session, rats were placed in conditioning boxes where the insertion of one lever (e.g., the left lever) signaled the delivery of sucrose to the food well and the other lever (e.g., the right lever) did not. This procedure allows the discriminative control by the two levers to be assessed, and thereby controls for a variety of changes in behavior that might not be a consequence of learning about the relationship between the lever and the reinforcer (in this case sucrose). The issue of principal interest was the link between lick cluster size (i.e., palatability) and individual differences in sign-tracking and goal-tracking. To assess this relationship, we examined the relationship between lick cluster size and the two target behaviors once a stable level of responding had been reached.

In a final test, rats were placed in the drinking chambers where they either received sucrose or water, and immediately after were placed in the operant boxes where they received presentations of the left and right lever but no sucrose. The issue of interest was whether devaluing sucrose in this way had effects on sign-tracking or goal-tracking (cf. Morrison et al., 2015). The results of this manipulation, at a group level, should provide converging evidence about the role of the current value of sucrose in determining the levels of sign- and goal-tracking.

### Method

#### Subjects

Sixteen naïve male (outbred) Lister Hooded rats (supplied by Harlan Laboratories, United Kingdom) were housed in pairs in standard cages and maintained on a 12-h/12-h light/dark cycle (lights on at 7 a.m.). Their mean ad libitum weight was 255 g (range: 228–279 g). During the first 5 days, rats had ad lib access to food and received 60-min access to water per day, 1 hr after the training sessions in which they were acclimated to the licking chambers. Rats were then given free access to water and they were maintained at between 85 and 90% of their ad lib weights by being given restricted access to food at the end of each day.

#### Apparatus

The experimental sessions occurred in two different rooms. The first room was that used in Experiment 1 and contained eight conditioning boxes that were placed in sound-attenuating shells. The second room contained six automated drinking chambers (Med Associates, Inc.) measuring 30 × 24 × 21 cm (H × W × D). The floor of each chamber was constructed from 19 steel rods: 4.8 mm diameter, 16 mm apart. The front wall, which served as the door, and back wall of each chamber were clear Perspex, and the other two walls were aluminum. The right-hand aluminum wall contained two apertures (2 × 1 cm) that were approximately 5 cm above the grid floor. The drinking tubes were mounted on retractable carriages that automatically positioned the spout level with the outside of the drinking aperture. At the end of each session, these tubes were automatically retracted. Only the left-hand spout was used in this experiment. The time of each lick was recorded by a contact-sensitive lickometer; and the tubes were weighed before and after each session to assess the amount consumed. The equipment was controlled by MED-PC software (Med Associates, Inc.), which also recorded the data. The fluids presented in these boxes were water during the acclimation sessions and sucrose (8%/water) during the experimental sessions. The key measure extracted from the record of licks was mean lick cluster size. A cluster was defined as a set of licks each separated by an interlick interval of a specified length of time. A number of different criteria have been adopted to define a new lick cluster, perhaps the most common are: 0.5 s used by Davis and his coworkers in developing this procedure (e.g., Davis & Perez, 1993; Davis & Smith, 1992; see also, Davies et al., 2015; Dwyer et al., 2008; Dwyer, Burgess, & Honey, 2012), 0.25 s (referred to as the “burst” rather than cluster criteria by Davis & Smith, 1992), and 1 s (e.g., Dwyer, Pincham, Thein, & Harris, 2009; Spector et al., 1998). Although the choice of criteria typically has little material influence on the results (as most pauses longer than 0.25 s are also longer than 0.5 or 1 s) we report the results for all three criteria here.

#### Procedure

##### Pretraining

Rats were first trained to drink from the tubes in the drinking chambers. On Days 1–5, they were placed in the drinking chambers for either 5 min (Day 1) or 15 min (Days 2–5). On the first day of pretraining, water was available upon being placed in the chamber, whereas on subsequent days there was a 60-s interval before the drinking tubes were introduced into the chamber. On the following day, rats were trained to consume sucrose from the food wells in the conditioning boxes in a 24-min session. During this session, 10-s presentations of sucrose were delivered on a VT 60-s (range: 40–80 s) schedule.

##### Palatability tests and conditioning

Rats received 5 × 5-day cycles that each consisted of a 15-min palatability test on one day (in the drinking chambers) followed by four, 53-min conditioning sessions on the following 4 days (in the conditioning boxes). The two types of session occurred at approximately the same time of day for a given rat. The palatability test sessions consisted of being placed in the licking chambers with access to 8% sucrose. The licking tubes were introduced into the chamber 60-s after the rats were placed in the chambers, and the pattern of licking was then recorded for the next 15 min whereupon the lick tubes were retracted. Consumption was recorded in the same way as during pretraining. When rats entered the conditioning boxes, the levers were retracted and the dippers were in a raised position but without any sucrose in them. The conditioning sessions consisted of 20 reinforced trials on which the lever was inserted for 10 s and its retraction was immediately followed by the dipper being immersed in 8% sucrose and then raised back into the food well. On the 20 nonreinforced trials, the second lever was presented and was followed by no programmed consequences. The order in which these two trial types was presented was random with the constraint that there were no more than three trials of the same type in succession; and the trials were delivered according to a VT 60-s (range: 40–80 s) schedule. For half of the rats, presentations of the left lever were followed by sucrose and those of the right lever were not, and for the remainder this arrangement was reversed.

##### Testing

The within-subjects devaluation procedure consisted of 3 days: Test cycle 1, Retraining, and Test cycle 2. On Test cycle 1, half of the rats were first placed in the licking chambers for 15 min and received access to 8% sucrose, and the remainder received water. The rats were then immediately transferred to the conditioning boxes, where they received a 27-min test session that consisted of 10 presentations of both of the levers neither of which was followed by sucrose. For half of the rats, the left lever had been followed by sucrose and for the remainder right lever had been paired with sucrose. On the second day, rats received a retraining session that was identical to the conditioning session that they had received during the training cycles. On Test cycle 2, the rats that had received water in Test cycle 1 received sucrose, and those that had received sucrose received water. The rats were then immediately placed in the conditioning boxes for the 27-min test session where they again received 10 separate presentations of both of the levers, and no sucrose was presented. Details of these test sessions that have not been mentioned were the same as during the conditioning training cycles.

### Results and Discussion

The 20 training sessions were combined into 10 consecutive two-day blocks. Bias scores were calculated for the final two blocks as in Experiment 1. These scores were then used to create two subgroups based on a median split: a goal-tracking group (high bias scores) and sign tracking group (low bias scores). The classifications were entirely congruent between Blocks 9 and 10 (i.e., the same eight rats were classified as goal-tracking on both blocks, while the remaining eight rats were classified as sign-tracking on both blocks, χ^2^ (1) = 16, *p* < .001, B_10_ > 1,000). Moreover, treating the bias scores as continuous variables (as in Experiment 1) revealed a strong positive correlation between scores on Blocks 9 and 10 (*r* = .88, *p* < .001, B_10_ > 1,000; descriptive data not shown).

**Figure 4 fig4:**
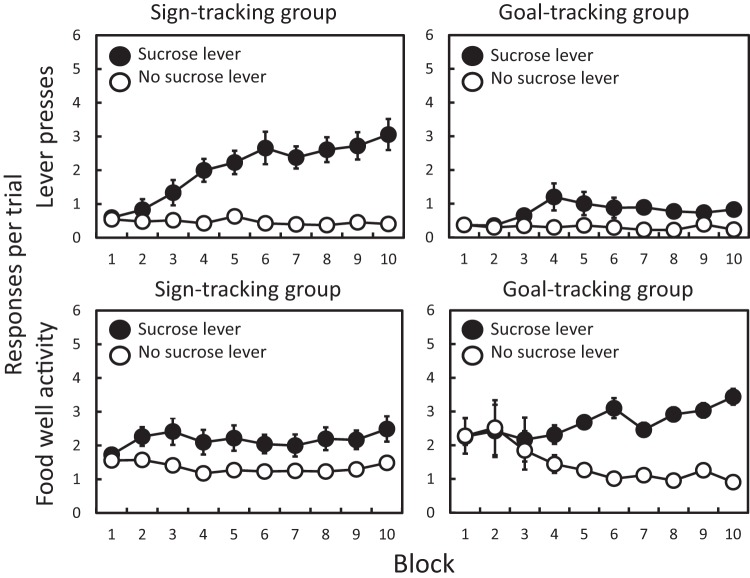
Experiment 2. Mean (±*SEM*) lever press responses (upper panels) and food well entries per trial (lower panels), across two-session blocks, for groups classified as sign-trackers (left panels) and goal-trackers (right panels). The scores within each panel are separated for the lever paired with sucrose (e.g., left lever) and the lever that was not paired with sucrose (e.g., right lever). Left and right levers were assigned to be reinforced and nonreinforced levers in a counterbalanced fashion.

The development of sign- and goal-tracking behavior is illustrated in Figure 4, with the upper panels showing lever press responses and the lower panels showing food well entries. Inspection of Figure 4 suggests that the difference in responding to the reinforced and nonreinforced levers was greater for lever pressing responses for the sign-tracking than the goal-tracking group, but that the reverse was true for food well entries. These results were analyzed using mixed ANOVAs with repeated measures factors of training block and lever (reinforced versus nonreinforced), and a between-subjects factor of sign- versus goal-tracking classification. The analysis was performed separately for lever press responses and food well entries.[Fig-anchor fig4]

For lever press responses (upper panels of Figure 4) there were significant main effects of lever (*F*(1, 14) = 52.64, *p* < .001, η_p_^2^ = .640), block (*F*(9, 126) = 10.51, *p* < .001, η_p_^2^ = .429) and classification (*F*(1, 14) = 11.04, *p* = .005, η_p_^2^ = .441). There was also a block-by-classification interaction (*F*(9, 126) = 5.05, *p* < .001, η_p_^2^ = .171) and lever-by-block interaction (*F*(9, 126) = 18.73, *p* < .001, η_p_^2^ = .463). Of most theoretical importance is the fact that both the classification-by-lever interaction (*F*(1, 14) = 15.56, *p* < .001, η_p_^2^ = .189) and the three-way interaction (*F*(9, 126) = 7.74, *p* < .001, η_p_^2^ = .191) were significant. Moreover, a Bayesian ANOVA comparison of the model based on the interaction to the best model without the interaction gives a Bayes factor of more than 1,000 in favor of the model with the interaction.

A parallel analysis of food well entries (lower panels of Figure 4) revealed a significant main effect of lever (*F*(1, 14) = 67.11, *p* < .001, η_p_^2^ = .827) but no main effects of block (*F*(9, 126) = 0.77, *p* = .641, η_p_^2^ = .052) or classification (*F*(1, 14) = 1.62, *p* = .280, η_p_^2^ = .083). There was a lever-by-block interaction (*F*(9, 126) = 12.66, *p* < .001, η_p_^2^ = .475), but no block-by-classification interaction (*F*(9, 126) = 0.27, *p* = .982, η_p_^2^ = .019). Although the classification-by-lever interaction was not significant (*F*(1, 14) = 2.79, *p* = .117, η_p_^2^ = .166), the three-way interaction was significant (*F*(9, 126) = 7.24, *p* < .001, η_p_^2^ = .341). In this case, the Bayesian ANOVA comparison of the model based on the interaction to the best model without the interaction gives a Bayes factor of 1.9 in favor of the model with the interaction.

**Figure 5 fig5:**
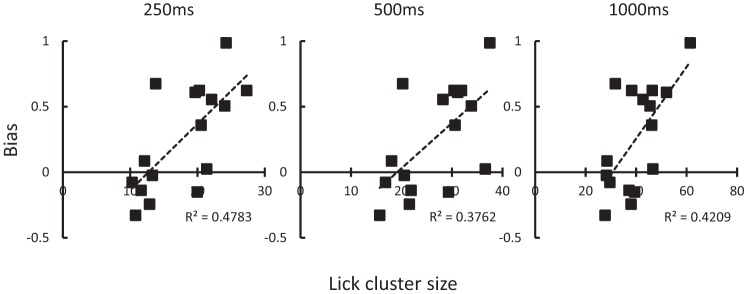
Experiment 2. The relationships between sign- versus goal-tracking bias and mean lick-cluster size (for the 0.25, 0.5, and 1 s pause criteria used to define new clusters).

The relationship between sucrose palatability scores (mean lick cluster size pooled over the four assessments) and the goal- versus sign-tracking bias score on the final blocks of training (9 and 10) is shown in Figure 5 (plotted separately for each of the three pause criterion used to define new lick clusters). Inspection of Figure 5 shows that there are strong positive correlations between palatability and goal-tracking bias for each of 3 commonly used pause criteria (*r* = .69, *p* = .003, B_10_ = 17.5; *r* = .61, *p* = .012, B_10_ = 5.8; *r* = .65, *p* = .007, B_10_ = 9.2; for 0.25, 0.5, and 1 s criteria, respectively). Moreover, when the palatability scores for the sign- and goal-tracking groups identified above are compared (see Table 2), the goal-tracking group displayed higher mean lick cluster sizes for the same three pause criteria, *t*(14) = 3.61, *p* = .003, *d* = 1.81, B_10_ = 13.2; *t*(14 = 2.60, *p* = .021, *d* = 1.30, B_10_ = 3.1; *t*(14) = 2.80, *p* = .014, *d* = 1.40, B_10_ = 4.1: for the 0.25, 0.5, and 1 s criteria, respectively). [Fig-anchor fig5][Table-anchor tbl2]

**Figure 6 fig6:**
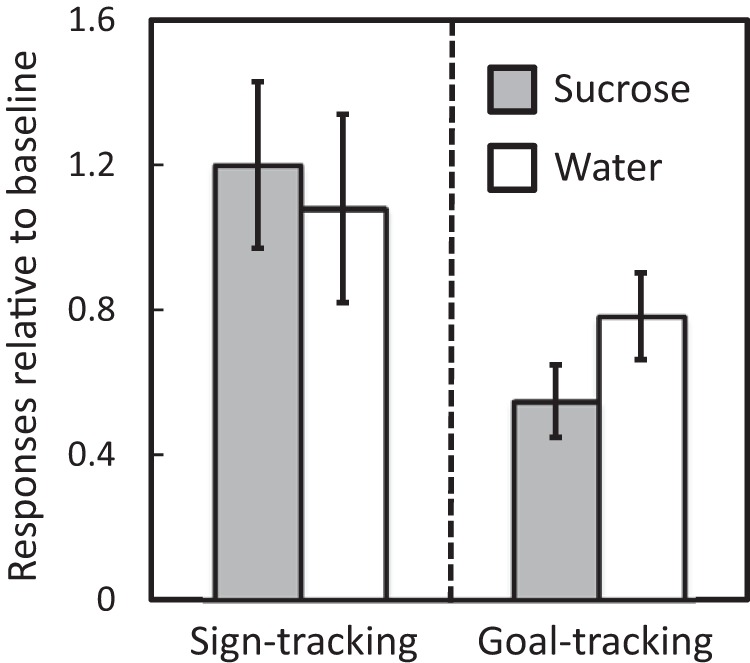
Experiment 2. Mean (±*SEM*) lever presses (left panel) and food well entries (right panel) relative to baseline (on Block 10 of training) after satiation with sucrose or access to water.

Finally, Figure 6 shows that, considered across all rats, while lever press behavior was not influenced by whether rats had been sated with sucrose or given access to water, *t*(15) = 0.55, *p* = .59, *d* = 0.28, B_01_ = 5.6: one-tailed, goal-tracking was reduced by sucrose satiation relative to access to water, *t*(15) = 2.22, *p* = .021, *d* = 0.56, B_10_ = 3.3: one-tailed. These results complement the relationship between individual differences in palatability and the goal-tracking bias evident in Figure 5: the palatability of sucrose is directly related to the goal-tracking bias and reducing the value of sucrose through satiation reduces goal-tracking, but not sign-tracking.[Fig-anchor fig6]

## General Discussion

Pavlovian conditioning is observed across the animal kingdom and is widely employed to study learning in laboratory settings. Part of its appeal is that it affords a high degree of control over the events to which the animal is exposed. Here then is a procedure that allows relationships to be arranged and their encoding to be observed through the “tracer” provided by variations in the conditioned response (Konorski, 1967). The study of Pavlovian conditioning has generated several well-established associative models that provide a detailed analysis of the mechanisms that underlie changes in learnt behavior (e.g., Mackintosh, 1975; Pearce & Hall, 1980; Rescorla & Wagner, 1972; Wagner, 1981). While these models can provide an account for both variations in the development and the form of conditioned behavior (e.g., Wagner & Brandon, 1989) they do not address the individual differences in sign- and goal-tracking studied here. Although such models could appeal to general individual differences (e.g., in sensory processes, states, or response repertoire), this appeal cannot provide an account for the signal-reinforcer selectivity of sign- and goal-tracking effects observed in Experiment 1: Such individual differences should be equally likely to affect responding during both levers, and yet individual differences on one lever (paired with one reinforcer) were not related to individual differences on the other lever (paired with a different reinforcer). At the same time, these results are inconsistent with the idea that differences in “temperament” are responsible for individual differences in sign- and goal-tracking. Instead the results of Experiment 2 provide direct support for the suggestion that the selectivity observed in Experiment 1 originates in the value of the reinforcer to the rat. If one accepts the general proposal that individual differences in reinforcer value interact with how learning is expressed (i.e., as goal- or sign-tracking) then what one needs is a principled account that can capture this interaction.

One model of learning that has separate processes that change during conditioning was described by Mackintosh (1975). This associative model assumes that conditioning trials result in the growth in an association between the signal and reinforcer (see also, Rescorla & Wagner, 1972), and an increase in attention to the signal. It is tempting to suggest that goal-tracking reflects the capacity of the signal to activate the representation of the reinforcer, through the associative link between them, and sign-tracking reflects attention to the signal based on its predictive history. These two types of process and their corresponding behavioral indices might be differentially affected by individual differences in palatability; with individual differences in palatability (or the effectiveness) of a reinforcer having an impact on performance (i.e., goal-tracking) both because it could affect the growth in the association and to the extent that the value of the reinforcer influences performance. However, the fact that goal-tracking and sign-tracking are, at least to some extent, incompatible behaviors, means that the influence of increasing palatability on goal-tracking might well reduce the behavioral expression of attention (i.e., sign-tracking); and sign-tracking might then appear to be more evident in animals for whom the reinforcer has less value. This analysis receives converging support from the results of Experiment 2, and those of Morrison et al. (2015), who showed that devaluing sucrose, by pairing it with lithium chloride, reduced goal-tracking and increased sign-tracking in goal-tracking rats.

The analysis outlined in the previous paragraph, in terms of associative and attentional processes, has the virtue of providing an account for the results of Experiment 2, where goal-tracking behavior was influenced by devaluation of the reinforcer, but sign-tracking was not: The strength of the associative link will interact with reduced value of the reinforcer to determine goal-tracking, but the predictive history of the signal (and sign-tracking) is left unchanged by the same devaluation treatment. However, this analysis appears to be undermined by the fact that procedures that degrade the predictive relationship between the stimulus and reinforcer, which should reduce both goal-tracking and sign-tracking, produce a reduction in goal-tracking but an increase in sign-tracking (e.g., Anselme, Robinson, & Berridge, 2013; Boakes, 1977). These observations are difficult to interpret: degrading the predictive relationship will reduce goal-tracking that, given the competing nature of the two responses, could increase sign-tracking as a secondary consequence (for further discussion, see Anselme, 2015).

An alternative analysis for the interaction between individual differences in reinforcer value and the development of sign- and goal-tracking appeals not to separate processes of association and attention, but to differences in the nature of the associations that develop during Pavlovian conditioning. It is popular to distinguish between associations that encode the relationship between a stimulus and a reinforcer, and stimulus–response associations that are simply stamped in by the reinforcer (e.g., Dickinson, 1980; see also, Holloway & Domjan, 1993). It seems possible that while goal-tracking reflects the formation of a stimulus-reinforcer association, sign-tracking develops because an approach response to the stimulus (e.g., the sight of the lever in this case; see Saunders & Robinson, 2012; Yager & Robinson, 2013) is stamped in by the presentation of the reinforcer (see also, Buzsaki, 1982; Lesaint, Sigaud, Flagel, Robinson, & Khamassi, 2014). Of course, one standard way to assess whether a given behavior is generated by stimulus-reinforcer or stimulus–response associations is through assessing its sensitivity to the current value of the reinforcer: If a behavior originates in a stimulus-reinforcer association it should be directly tied to the current value of the reinforcer, but if it reflects the development of a stimulus-response association, reinforcer devaluation should not affect performance. This distinction, therefore, provides a ready account for the fact that changes in the value of the reinforcer affect goal-tracking, but not sign-tracking (Experiment 2; see also, Morrison et al., 2015). This is not to deny the possibility that under some conditions, perhaps when rats have received more limited training (see Adams, 1982; Adams & Dickinson, 1981), changing the value of the reinforcer previously associated with a lever signal might affect sign-tracking (cf. Robinson & Berridge, 2013).

We have shown that it is possible characterize individual differences in how learning is expressed in terms of general-process accounts of associative learning by considering the role of individual differences in reinforcer value. However, what is the origin of these individual differences in reinforcer value? One possibility is that these differences in value (and in goal-tracking) are related to variations in dopamine release. However, Flagel et al. (2011) showed that the dopamine response provoked by a reinforcer transfers to the signal in sign-trackers, but not goal-trackers (see also, Anselme et al., 2013). Moreover, the finding that different reinforcers from the same motivational system do not produce corresponding patterns of sign-tracking (or goal-tracking) within a given rat (Experiment 1), is inconsistent with the idea that different appetitive reinforcers (food pellets and sucrose) share the same value within the “common currency” of dopamine release. Of course, it remains possible that these appetitive reinforcers generate individual differences in dopamine release (see Cameron, Wightman, & Carelli, 2014; McCutcheon, Beeler, & Roitman, 2012). Nevertheless, it is of interest to note that rats that have been selectively bred to prefer alcohol are more likely to engage in goal-tracking, and less likely to engage in sign-tracking, than their nonalcohol preferring counterparts (Peña-Oliver et al., 2015). These results suggest an important link between a propensity to prefer alcohol and goal-tracking based on a food reinforcer. The neurobiological bases of individual differences in reinforcer value, therefore, represent an important target for further investigation.

We conclude that the key novel observations from Experiments 1 and 2 were that individual differences in sign- and goal-tracking (a) were not consistent across different signal-reinforcer relationships, and (b) were mediated by reinforcer value. These observations provide a foundation upon which general-process models of associative learning can explain the origin of marked differences in how learning is expressed during Pavlovian conditioning.

## Figures and Tables

**Table 1 tbl1:** Experiment 1: Consistency of Sign-Tracking and Goal-Tracking Classifications

Classifications		Sign-tracking rats	Goal-tracking rats
A: Left lever on Blocks 5 and 6		Split by the bias on the left lever on Block 6	
Split by the bias on the left lever on Block 5	Sign-tracking rats	13	3
	Goal-tracking rats	3	13
B: Right lever on Blocks 5 and 6		Split by the bias on the right lever on Block 6	
Split by the bias on the right lever on Block 5	Sign-tracking rats	14	2
	Goal-tracking rats	2	14
C: Left and right levers on Block 5		Split by bias on the right lever on Block 5	
Split by the bias on the left lever on Block 5	Sign-tracking rats	9	7
	Goal-tracking rats	7	9
D: Left and right levers on Block 6		Split by the bias on the right lever on Block 6	
Split by the bias on the left lever on Block 6	Sign-tracking rats	8	8
	Goal-tracking rats	8	8
*Note*. Sections A and B show the consistency between sign- and goal-tracking classifications on the left lever on Blocks 5 and 6 (A), and the right lever on Blocks 5 and 6 (B). Sections C and D show the lack of consistency between sign- and goal-tracking classifications on the left and right levers on Block 5 (C) and Block 6 (D).			

**Table 2 tbl2:** Experiment 2: Mean Lick Cluster Size (for .25, .5, and 1 s Pause Criterion) for the Sign- and Goal-Tracking Sub-Groups

	Pause criterion		
Sub-group	.25 s	.5 s	1 s
Sign-tracking	14.0 (1.5)	22.6 (2.5)	34.4 (2.5)
Goal-tracking	21.5 (1.4)	30.5 (1.8)	45.5 (3.1)

## Footnotes

1 Bayes factors were calculated using hypergeometric sampling appropriate to the 2 × 2 contingency tables cases examined here (see Gûnel & Dickey, 1974).

2 Bayes factors were calculated for factorial ANOVAs in the way described by Rouder, Morey, Speckman, and Province (2012) and Rouder, Morey, Verhagen, Swagman, and Wagenmakers (2016).
